# Inhibition of Glycogen Synthase Kinase-3β Attenuates Glucocorticoid-Induced Suppression of Myogenic Differentiation *In Vitro*

**DOI:** 10.1371/journal.pone.0105528

**Published:** 2014-08-15

**Authors:** Zhenyu Ma, Zhigang Zhong, Zhenyang Zheng, Xing-Ming Shi, Weixi Zhang

**Affiliations:** 1 Department of Neurology, the First Affiliated Hospital, Sun Yat-sen University, Guangzhou, Guangdong Province, China; 2 Institute of Molecular Medicine and Genetics, Georgia Regents University, Augusta, Georgia, United States of America; University of Ulm, Germany

## Abstract

Glucocorticoids are the only therapy that has been demonstrated to alter the progress of Duchenne muscular dystrophy (DMD), the most common muscular dystrophy in children. However, glucocorticoids disturb skeletal muscle metabolism and hamper myogenesis and muscle regeneration. The mechanisms involved in the glucocorticoid-mediated suppression of myogenic differentiation are not fully understood. Glycogen synthase kinase-3β (GSK-3β) is considered to play a central role as a negative regulator in myogenic differentiation. Here, we showed that glucocorticoid treatment during the first 48 h in differentiation medium decreased the level of phosphorylated Ser9-GSK-3β, an inactive form of GSK-3β, suggesting that glucocorticoids affect GSK-3β activity. We then investigated whether GSK-3β inhibition could regulate glucocorticoid-mediated suppression of myogenic differentiation *in vitro*. Two methods were employed to inhibit GSK-3β: pharmacological inhibition with LiCl and GSK-3β gene knockdown. We found that both methods resulted in enhanced myotube formation and increased levels of muscle regulatory factors and muscle-specific protein expression. Importantly, GSK-3β inhibition attenuated glucocorticoid-induced suppression of myogenic differentiation. Collectively, these data suggest the involvement of GSK-3β in the glucocorticoid-mediated impairment of myogenic differentiation. Therefore, the inhibition of GSK-3β may be a strategy for preventing glucocorticoid-induced muscle degeneration.

## Introduction

Duchenne muscular dystrophy (DMD) is an incurable disease that manifests in children and is caused by mutations in the dystrophin gene [1]. Currently, glucocorticoids are the only available treatment that has been approved for clinical trials to improve muscle strength and function in affected children [2]. However, the harsh side effects of long-term glucocorticoid treatment, such as skeletal muscle atrophy and bone loss, limit their therapeutic application in clinical practice. In addition, various studies have highlighted the direct detrimental effect of glucocorticoid treatment on satellite cell function [3]–[5]. It was suggested that the lowered activation of adult muscle satellite cells and reduced myogenesis might partly account for the reduced skeletal muscle recovery from injury that is caused by glucocorticoid treatment [4], [6].

Satellite cells are muscle precursor cells that are anatomically located under the basal lamina of muscle fibers. Satellite cells play a critical role in muscle growth, homeostasis and regeneration [7]. Myogenic differentiation is a precisely controlled process in which myoblasts first withdraw from the cell cycle, differentiate and then finally fuse to form multinucleated myotubes [8]. In DMD, damaged or necrotic fibers are removed by inflammatory cells and repaired by satellite cells. Because of the fragility of the sarcolemma, newly generated fibers tend to cause further damage, and they undergo repeated degeneration-regeneration cycles that eventually result in the defective regenerative capacity of satellite cells [9]. Any conditions that impair satellite cell function could lead to an exacerbation of muscle pathology over the long term. Although glucocorticoids have been reported to suppress the myogenic differentiation of satellite cells, the mechanisms are poorly understood. Revealing these mechanisms could help us enhance the efficacy of glucocorticoid treatment in DMD by preventing its adverse effects on myogenic differentiation.

Glycogen synthase kinase-3β (GSK-3β) was originally identified as a suppressor of glycogen synthase. GSK-3β was later found to be ubiquitously expressed and implicated in multiple biological processes, including cell survival, cell differentiation and metabolic responses [10], [11]. Unlike most kinases, GSK-3β is maintained in an active state in resting cells and is inhibited by various growth factors via the phosphorylation of serine 9 [12]. In skeletal muscle, GSK-3β has been identified as a negative modulator of myogenic differentiation and adult muscle growth. The genetic or pharmacological inhibition of GSK-3β was reported to promote myogenic differentiation and reverse muscle atrophy [12]–[15].

Glucocorticoids exert their extensive effects in many pathological conditions by activating GSK-3β. The adverse effects of glucocorticoids on bone formation are mainly caused by increased levels of active GSK-3β [16]. A recent report showed that glucocorticoids lowered hippocampal neurogenesis via the GSK-3β/β-catenin signaling pathway, and leptin counteracted this suppression by inactivating GSK-3β via phosphorylation of serine 9 [17]. In particular, the atrophic effect of glucocorticoids on skeletal muscle is thought to be mediated in part by GSK-3β, which reduces the protein synthesis rate by inhibiting eukaryotic transcription factor 2B-dependent translation [18]. In the present study, we investigated whether GSK-3β was involved in the glucocorticoid-mediated suppression of myogenic differentiation *in vitro*. Furthermore, we examined whether the glucocorticoid-induced impairment of myogenic differentiation could be counteracted through the pharmacological and genetic inhibition of GSK-3β.

## Materials and Methods

### Materials

C57BL/6J mice (7 weeks) were purchased from the Laboratory Animal Center of Sun Yat-sen University (Guangzhou, China). All animal procedures were approved by the Institutional Animal Care and Use Committee (IACUC) at the Sun Yat-sen University.

The murine skeletal muscle cell line C2C12 was purchased from the American Type Culture Collection (ATCC, Manassas; USA). High glucose Dulbecco's Modified Eagle's Medium (DMEM), fetal bovine serum (FBS), horse serum (HS), collagen I, collagenase II and OPTI-MEM I Reduced Serum Medium were purchased from Gibco (Invitrogen Inc., Carlsbad, CA, USA). Dispase was purchased from Roche (Mannheim, Germany). Skeletal Muscle Cell Medium (SkMCM) was purchased from ScienCell Research Laboratories (Carlsbad, CA, USA). Dexamethasone, lithium chloride (LiCl) and Percoll were purchased from Sigma-Aldrich (St. Louis, MO, USA). RU-486 was purchased from Selleck Chemicals (Houston, TX, USA). TRIZOL Reagent and Lipofectamine 2000 Reagent were purchased from Invitrogen (Carlsbad, CA, USA). PrimeScript RT reagent kit and SYBR Premix Ex Taq were purchased from Takara (Dalian, China). A CCK-8 kit was purchased from Dojindo (Kumamoto, Japan). Anti-MyoD polyclonal antibody and anti-PAX-7 polyclonal antibody were purchased from Abcam (Cambridge, MA, USA); anti-myogenin (F5D) monoclonal antibody and myosin heavy chain (MF20) were purchased from the Developmental Studies Hybridoma Bank (University of Iowa, Iowa City, IA, USA); anti-β-tubulin monoclonal antibody was purchased from Sigma-Aldrich (St. Louis, MO, USA); and anti-GSK-3β polyclonal antibody, anti-phospho-GSK-3-beta (Ser9) polyclonal antibody and anti-cleaved-caspase-3 monoclonal antibody were purchased from Cell Signaling Technology (Beverly, MA, USA).

### Cell culture and treatments

Primary murine satellite cells were isolated from 7-week-old C57BL/6J mice using a modification of the protocol described by Zhang [19]. Briefly, skeletal muscles were digested with 0.2% collagenase II/2.4 U dispase in 1× PBS plus 1% penicillin/streptomycin at 37°C for 60 min on a rocker. The mixture was filtered through a 70-µm nylon mesh cell strainer and then subjected to Percoll gradient centrifugation. Primary murine satellite cells were obtained from the interface between 30% and 70% Percoll and were preplated into a collagen I–coated tissue culture flask twice for 2 h and then twice for 24 h to remove fibroblasts. The primary satellite cells were cultured in growth medium (Skeletal Muscle Cell Medium, SkMCM) or differentiation medium (DM; high glucose DMEM, 2% HS) in a humidified incubator (37°C with 5% CO_2_).

C2C12 cells were cultured in growth medium (high glucose DMEM, 10% FBS) or differentiation medium (DM; high glucose DMEM, 2% HS) in a humidified incubator (37°C with 5% CO_2_).

To induce myogenic differentiation by growth factor withdrawal, the growth medium was replaced with DM. The indicated concentrations of dexamethasone, lithium chloride (LiCl) or RU-486 were added directly after the induction of myogenic differentiation and again 24 h later, when the cells were provided with fresh DM. Cells from different conditions were harvested for the following experiments at the indicated time points.

### shRNA interference and transfections

Plasmids expressing short hairpin RNA (shRNA) specific for the GSK-3β gene and negative control shRNA plasmids were purchased from GenePharma Co. (Shanghai, China). The transfection was performed according to the manufacturer's instructions for Lipofectamine 2000 Reagent. Briefly, myoblasts were plated at 1×10^4^ cells/cm^2^ and cultured in growth medium to reach 80–90% confluence. The cells were washed twice with OPTI-MEM I Reduced Serum Medium and were then incubated with a mixture of DNA, Lipofectamine 2000 and serum-free medium for 5 h; then, the medium was replaced with growth medium. Twenty-four hours after transfection, the cells were harvested, and the silencing efficiency was determined by Western blot. In our experimental system, the plasmid containing the target sequence 5′-GGAGAGCCCAATGTTTCATAT-3′ demonstrated the strongest RNA silencing for GSK-3β.

### Real-time PCR analysis

The mRNA levels of MCK and GAPDH were determined by real-time PCR. PCR primers were synthesized by Life technologies (Shanghai, China). Total RNA from C2C12 cells was extracted using TRIZOL Reagent. Reverse transcription was performed using the PrimeScript RT reagent Kit using 500 ng of total RNA. The cDNA was frozen at −20°C and used in real-time PCR. The mRNA levels of MCK and GAPDH were detected using SYBR Premix Ex Taq (Perfect Real Time). All of the procedures were performed strictly according to the manufacturer's instructions. Standard curves were generated in duplicate by performing serial dilutions of pooled cDNA aliquots. The specificity of the reaction was confirmed by melting curve analysis. MCK transcript concentration for each sample was determined using a standard curve and compared to the untreated control samples. Analytical data were normalized to the mRNA expression level of GAPDH as an internal control. The primers used in this study are described in Table 1.

**Table 1:** Real-time PCR primer sequences used in this study.

Genes	Forward	Reverse
*MCK*	CTGACCCCTGACCTCTACAAT	CTGACCCCTGACCTCTACAAT
*GAPDH*	ACCCAGAAGACTGTGGATGG	ACACATTGGGGGTAGGAACA

### Western blot analysis

Cells were washed with cold PBS, and the cells were lysed by adding 1× SDS sample buffer. Afterwards, the cells were scraped immediately and sonicated for 10–15 sec. The samples were boiled for 5 min at 95°C and stored at −20°C. Equal amounts of protein extracts were separated on SDS-PAGE gels, followed by 0.45 µm PVDF membrane transfer (Millipore, Billerica, MA, USA) and immunoblotting with the desired primary antibodies at 4°C for 12 h. This was followed by incubation with horseradish-peroxidase-conjugated secondary antibody (1∶5000) at room temperature for 1 h. The antibody dilutions used were as follows: 1∶200 for MyoD1, 1∶300 for F5D, 1∶500 for MF20, 1∶1000 for GSK-3β and pho-Ser9-GSK-3β, 1∶1000 for β-tubulin, 1∶500 for cleaved-caspase-3. Blots were developed using the Immobilon Western Chemiluminescent HRP Substrate (Millipore, Billerica, MA, USA). The results were quantified using the Image J2.1.4.7 software.

### Immunofluorescence and myogenic differentiation assays

The cells were washed twice with cold PBS, fixed with 4% formaldehyde, permeabilized with 0.3% Triton X-100 in PBS and blocked in blocking buffer (1×PBS/5% normal goat serum/0.3% Triton X-100). The cells were incubated with diluted MF20 (1∶100) or PAX-7 (1∶50) overnight at 4°C. DyLight 594 goat anti-mouse IgG or Alexa Fluor 488-conjugated anti-rabbit IgG (H+L) were used as secondary antibodies (1∶400). The nuclei were counterstained with DAPI (20 µg/ml). Images were taken at 100× magnification using an Olympus digital camera.

To evaluate myogenic differentiation, a fusion index was calculated to indicate myotube fusion. For each condition, the total number of nuclei and the number of nuclei incorporated into the myotubes were counted using 10 images from randomly chosen microscope fields populated with DAPI- and MyHC-stained cells. The fusion index was calculated as the percentage of nuclei incorporated into myotubes (defined as containing at least two nuclei) relative to the total number of nuclei.

### Cytotoxicity assays

One hundred microliters of cell suspension was dispensed into each well of 96-well plates (5000 cells/well) and pre-incubated with growth medium for 24 h. The cells were then switched to DM supplemented with 0 M or 10^−5^ M DEX and cultured for 12 h, 24 h, and 48 h before they were harvested. Ten microliters of CCK-8 solution was added to each well, and the plate was incubated for 4 h before measuring the absorbance at 450 nm.

### Statistical analysis

All results were expressed as the mean ± SEM. The difference between groups was analyzed using Student's paired *t*-test (SPSS 18.0 software package). A two-tailed *P*<0.05 was considered statistically significant.

## Results

### Dexamethasone inhibits myogenic differentiation via the glucocorticoid receptor (GR)

**Figure 1:**
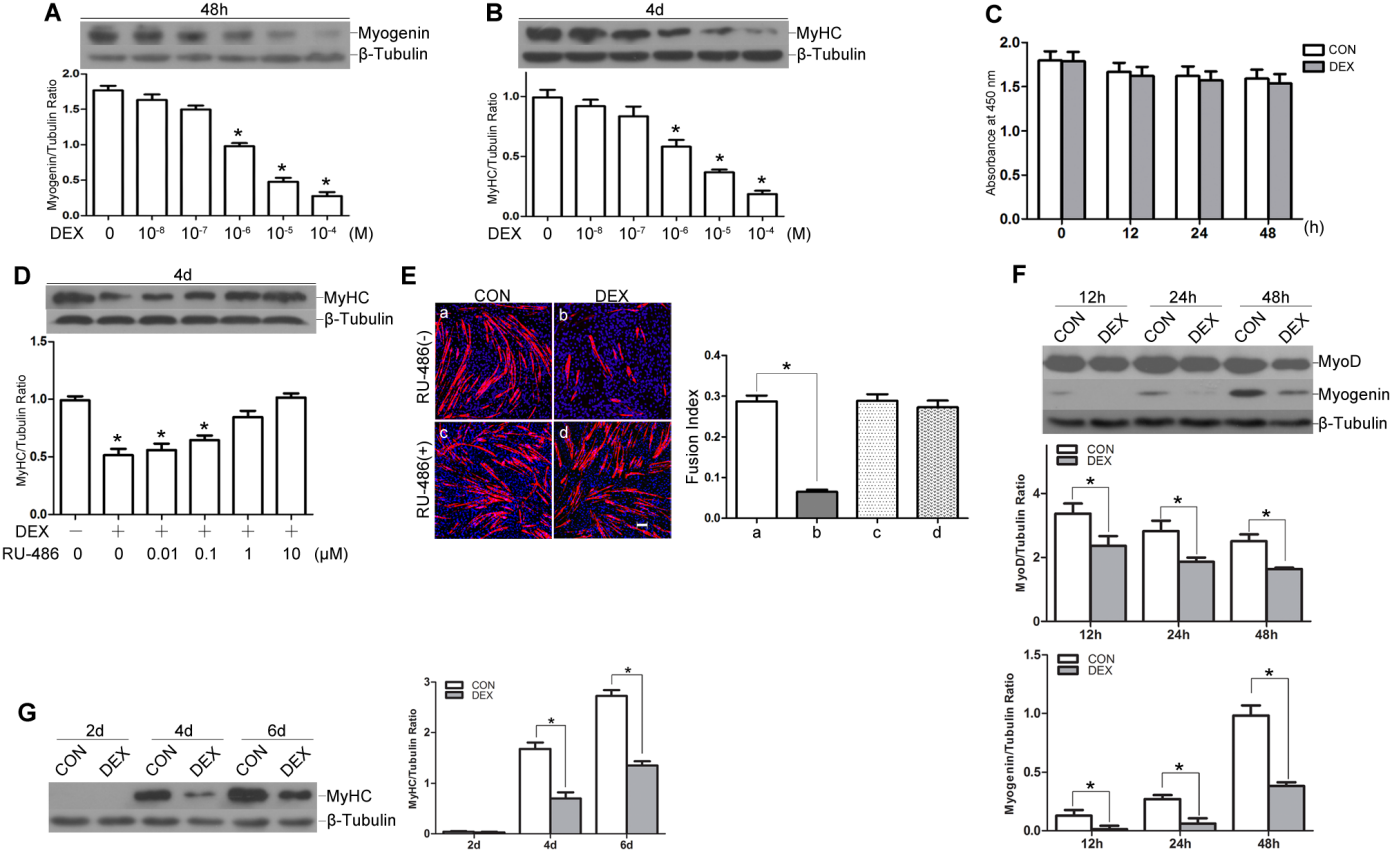
DEX inhibits myogenic differentiation via the glucocorticoid receptor (GR). (A and B) Differentiating C2C12 myoblasts were treated with various concentrations of DEX (0 to 10^−4^ M) for 48 h or 4 d. Western blot analysis showing the protein expression levels of myogenin at 48 h (A) and MyHC at 4 d (B). (*P<0.05 versus cells without DEX treatment; n = 3 independent experiments). (C) Differentiating C2C12 myoblasts were treated with 10^−5^ M DEX and harvested for cytotoxicity assays using a CCK-8 kit at 12 h, 24 h, and 48 h. (D) Application of a GR antagonist (RU-486) nearly restored the protein expression level of MyHC in a dose-dependent manner. (*P<0.05 versus cells without DEX and RU-486 treatments; n = 3 independent experiments). (E) Myogenic differentiation assay to determine the GR specificity of DEX by using RU-486 (10 µM). Immunofluorescence detection of MyHC (red) and DAPI counterstaining of nuclei (blue) was used to detect myotubes (left panel). The fusion index is shown in right panel. The scale bar is 50 µm. (F and G) Time-course experiments of DEX-induced suppression of myogenic differentiation. Western blot analysis of the early stage markers MyoD and myogenin at 12 h, 24 h, and 48 h (F) and the late stage marker MyHC at 2 d, 4 d, and 6 d (G). The data are shown as the means ± SEM of three independent experiments. *P<0.05.

To determine the effects of glucocorticoids on myogenic differentiation, we employed the established C2C12 myoblasts as an *in vitro* myogenic differentiation model. Differentiating myoblasts were treated with various concentrations of dexamethasone (DEX) (10^−8^ to 10^−4^ M) for 48 h or 4 d, and myogenic differentiation was determined by Western blot analysis of the expression levels of the early myogenic marker myogenin and the late marker MyHC. As shown in Fig. 1A and 1B, DEX reduced C2C12 myogenic differentiation in a dose-dependent manner compared with control myogenic induction. According to the results, treatment with 10^−5^ M DEX, a concentration that is widely used to analyze glucocorticoid-induced suppression of myogenic differentiation [3], [5], was chosen in the subsequent experiments.

We first determined the cytotoxicity of 10^−5^ M DEX to differentiating myoblasts. DEX time-course (0 to 48 h) experiments were performed, and the results showed that DEX at 10^−5^ M had no significant toxic effect on differentiating myoblasts (Fig. 1C).

We then evaluated the effects of the glucocorticoid receptor (GR) antagonist RU-486 on myogenic differentiation to study the mechanistic action of DEX. Increasing concentrations of RU-486 (from 0.01 µM to 10 µM) prevented the inhibitory effects of DEX on myogenic differentiation in a dose-dependent manner, and RU-486 (10 µM) alone showed no effects on myogenic differentiation (Fig. 1D and 1E). These results revealed that the inhibitory effects of DEX on myogenic differentiation were mediated through a steroidal pathway via the GR.

To fully evaluate the inhibitory effects of glucocorticoids on myogenic differentiation, we performed DEX time-course experiments. The results showed that incubation with DEX led to a significant reduction of myogenin expression and a mild reduction of MyoD expression during first 48 h, an early differentiation stage (Fig. 1F). At 4 d and 6 d after differentiation, which are the terminal stages of myogenic differentiation, the MyHC protein levels decreased in response to DEX treatment (Fig. 1G).

### Effects of DEX on the phosphorylation of GSK-3β during myogenic differentiation

**Figure 2:**
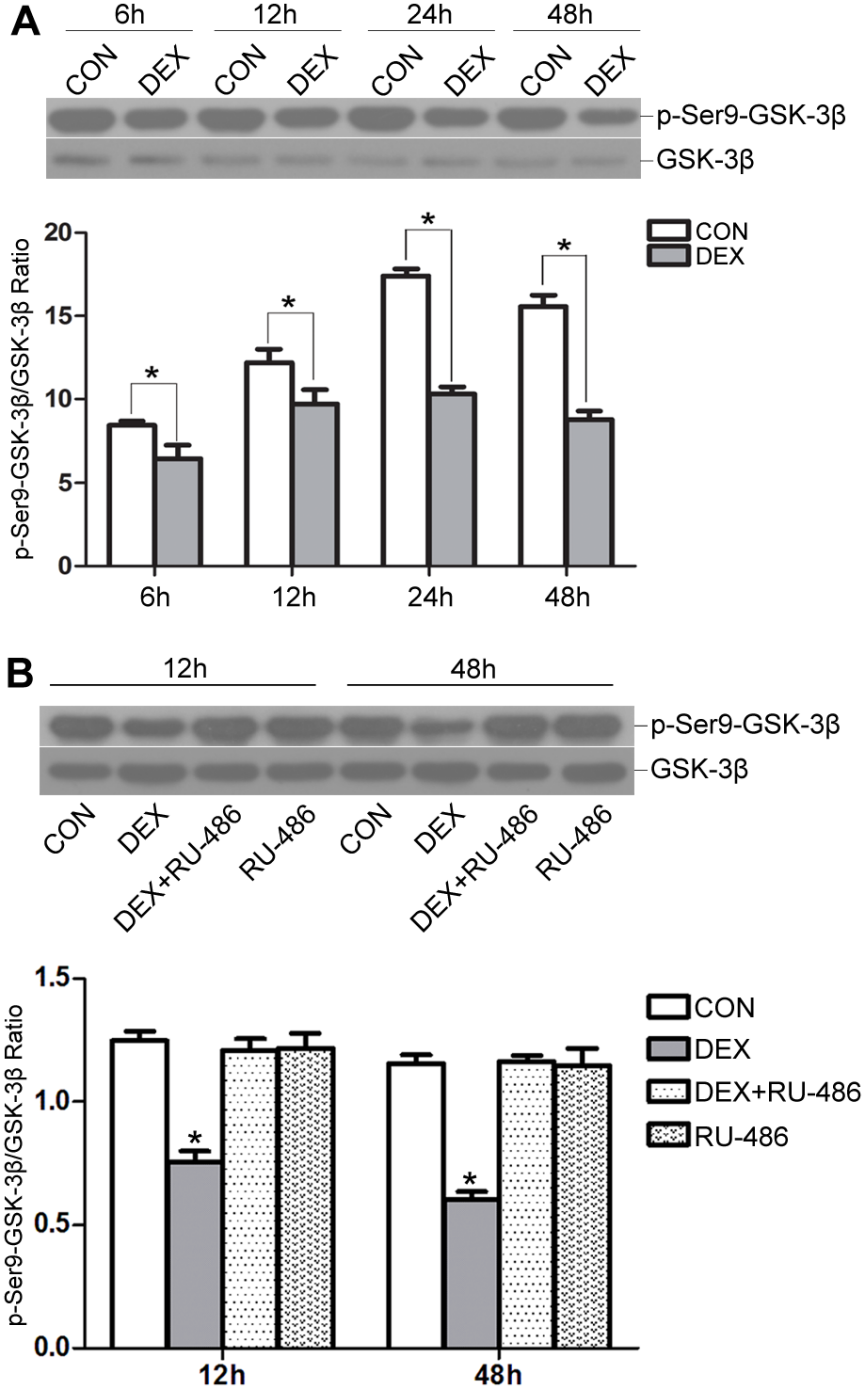
Effects of DEX on GSK-3β activity during myogenic differentiation. (A) C2C12 myoblasts were induced to differentiate in the absence (CON) or presence of 10^−5^ M DEX. Phosphorylation of GSK-3β at serine 9 at the indicated time points was determined using Western blot analysis. (B) Differentiating C2C12 myoblasts were incubated with DEX and RU-486 (10 µM), alone or in combination, or control DM (CON). Phosphorylation of GSK-3β at serine 9 at the indicated time points was determined using Western blot analysis. The data are shown as the means ± SEM of three independent experiments. *P<0.05 compared to control group (without DEX or RU-486 in DM).

Next, we evaluated whether DEX treatment affected the phosphorylation of GSK-3β during the induction of myogenic differentiation. As shown in Fig. 2A, the addition of DEX to differentiating C2C12 myoblasts at an early stage of differentiation caused the reduced phosphorylation of GSK-3β on serine residue 9 and resulted in an inactive form of GSK-3β. In addition, application of RU-486 ablated the active effects of DEX on GSK-3β activity (Fig. 2B). These results indicate that DEX addition during myogenic differentiation could increase GSK-3β activity via the GR *in vitro*.

### Pharmacological inhibition of GSK-3β rescues the impaired myogenic differentiation caused by dexamethasone

**Figure 3:**
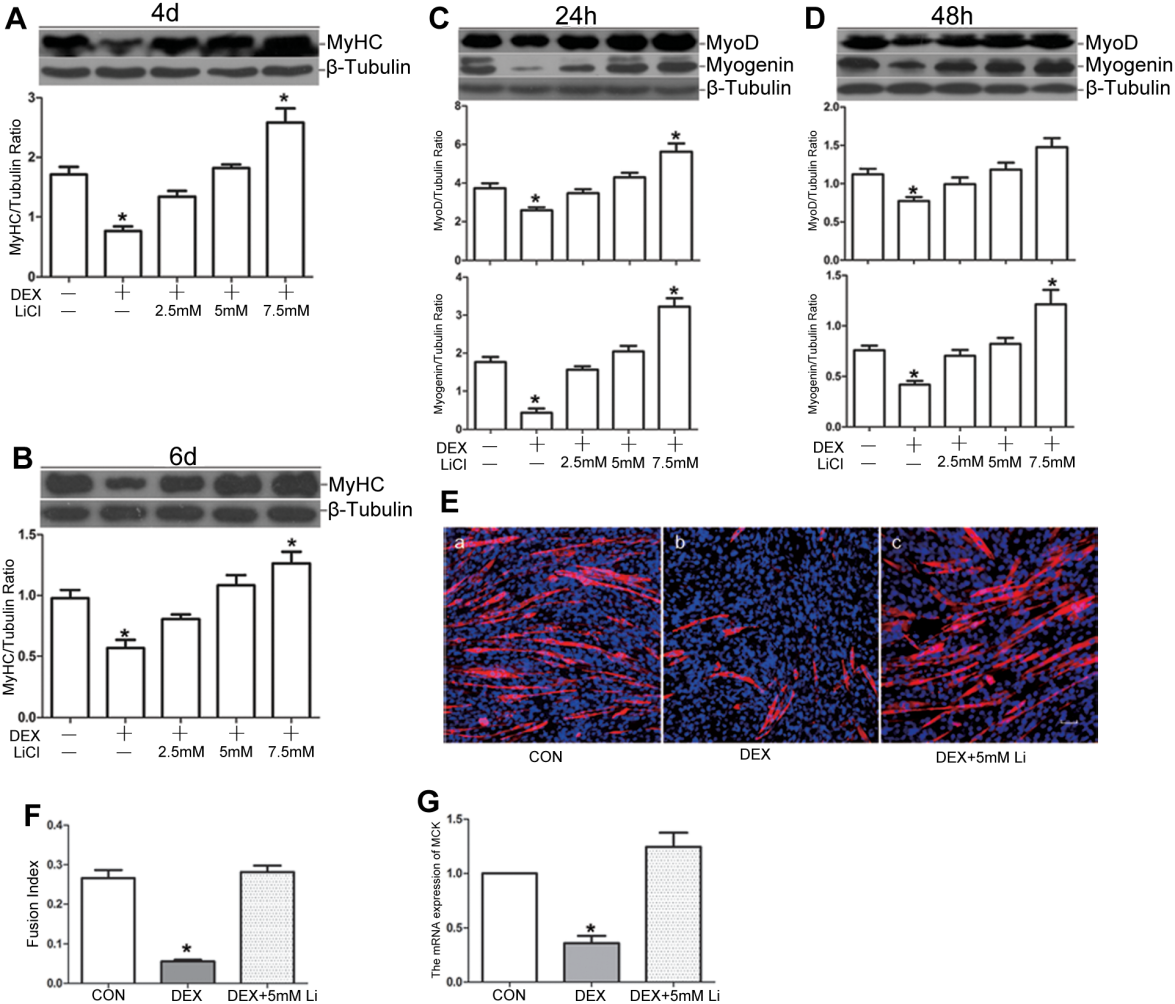
Pharmacological inhibition of GSK-3β rescues the impaired myogenic differentiation caused by dexamethasone. C2C12 myoblasts were treated with DEX (10^−5^ M) or a combination of DEX and various concentrations of LiCl (2.5, 5, 7.5 mM) after switching to DM. (A and B) Western blot analysis of MyHC protein expression when induced for 4 d (A) and 6 d (B). (C and D) Western blot analysis of MyoD and myogenin expression at the early stage of myogenic differentiation, *i.e.*, induced for 24 h (C) and 48 h (D). (E) C2C12 myoblasts were treated with DEX (10^−5^ M) or a combination of DEX and LiCl (5 mM) and induced to differentiate for 4 d. Immunofluorescence detection of MyHC (red) and DAPI counterstaining of nuclei (blue) were used to label myotubes. The scale bar is 50 µm. (F) Quantitative analysis of the fusion index from (E). (G) C2C12 myoblasts were induced to differentiate for 3 d in the presence of DEX (10^−5^ M) or a combination of DEX and LiCl (5 mM), and MCK mRNA levels were measured by real-time RT-PCR and compared to control myoblasts (without DEX or LiCl in DM). The data are shown as the means ± SEM of three independent experiments. *P<0.05 versus the control group (without DEX or LiCl in DM).

Differentiating C2C12 myoblasts were treated with 10^−5^ M DEX together with various concentrations of LiCl (0, 2.5, 5, or 7.5 mM), which is an established and widely used inhibitor of GSK-3β [20], [21]. We first performed Western blot analysis using whole-cell lysates to determine the protein expression pattern of MyHC. As shown in Fig. 3A and 3B, the reduced expression of MyHC at 4 d and 6 d was ameliorated by co-treatment with DEX and LiCl in a concentration-dependent manner, and 5 mM LiCl could nearly reverse MyHC expression to normal level. At early differentiation stages, LiCl exerted an antagonistic effect on DEX during myogenic differentiation, as indicated by the expression of MyoD and myogenin (Fig. 3C and 3D). To further confirm the antagonistic effect of LiCl, immunofluorescent staining of MyHC was performed for morphological analysis. After 4 d of differentiation induction, myoblasts co-treated with 5 mM LiCl and DEX presented similar myotube formation and fusion index with control group, which reconfirmed the antagonistic effect of LiCl on the anti-myogenic effect of DEX (Fig. 3E and 3F). Furthermore, we observed a significant DEX-mediated down-regulation of MCK mRNA expression compared to the control myoblasts after a 3-d incubation in DM. In contrast, the inhibition of GSK-3β with 5 mM LiCl strongly up-regulated MCK mRNA expression to normal level, despite the presence of DEX in the DM (Fig. 3G).

### GSK-3β knockdown attenuates dexamethasone-induced repression of myogenic differentiation

**Figure 4:**
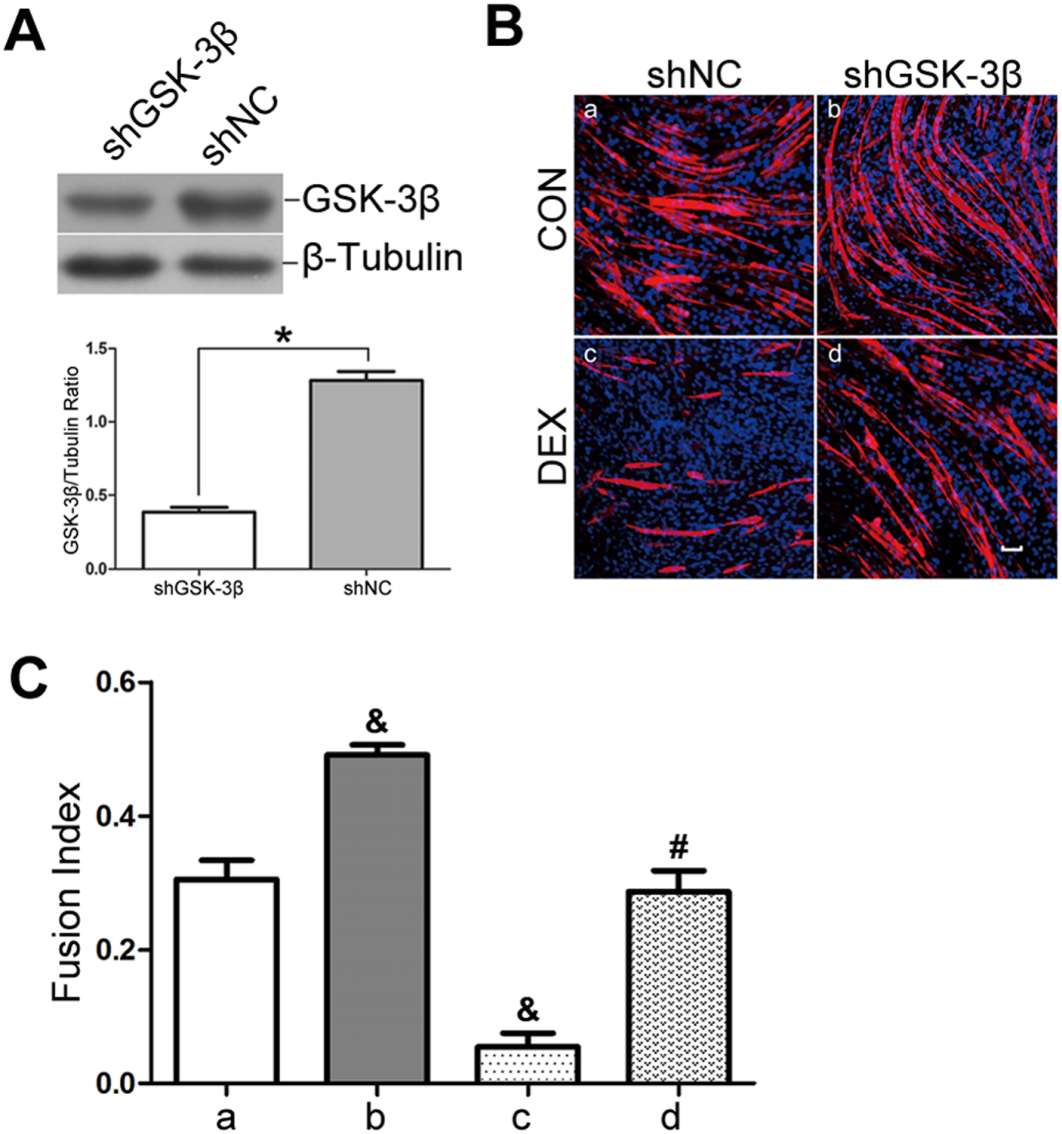
GSK-3β knockdown attenuates DEX-induced impairment of myotube formation. (A) shRNA interference of GSK-3β was performed in C2C12 myoblasts, and GSK-3β abundance was assessed by Western blot analysis to detect the silencing efficiency after transfection for 24 h. (B) shNC myoblast cells and shGSK-3β myoblast cells were differentiated in the absence (CON, panel a, b) or presence of DEX (DEX, panel c, d) for 4 d. Immunofluorescence detection of MyHC (red) and DAPI (blue) were used to detect myotubes. The scale bar is 50 µm. (C) Quantitative analysis of the fusion index using data from (B). The data are shown as the means ± SEM of three independent experiments. *P<0.05; ^&^ *P*<0.05 versus shNC/CON; ^#^ *P*<0.05 versus shNC/DEX.

Plasmids expressing shRNA were employed to specifically knockdown GSK-3β mRNA in C2C12 myoblasts. In myoblasts transfected with GSK-3β shRNA (shGSK-3β), a 70% decline in GSK-3β protein abundance was observed compared with the negative control shRNA (shNC)-transfected myoblasts 24 h after transfection (Fig. 4A).

After a 4-d differentiation in the absence of DEX, we observed that the shGSK-3β myoblasts exhibited robust myotube formation compared with shNC myoblasts using MyHC staining and fusion index analysis (Fig. 4B, upper panel; Fig. 4C). Importantly, GSK-3β knockdown attenuated the inhibitory effects of DEX on myogenic differentiation as shown by MyHC staining and a similar fusion index compared with shNC myoblasts in control condition (Fig. 4B; Fig. 4C).

**Figure 5:**
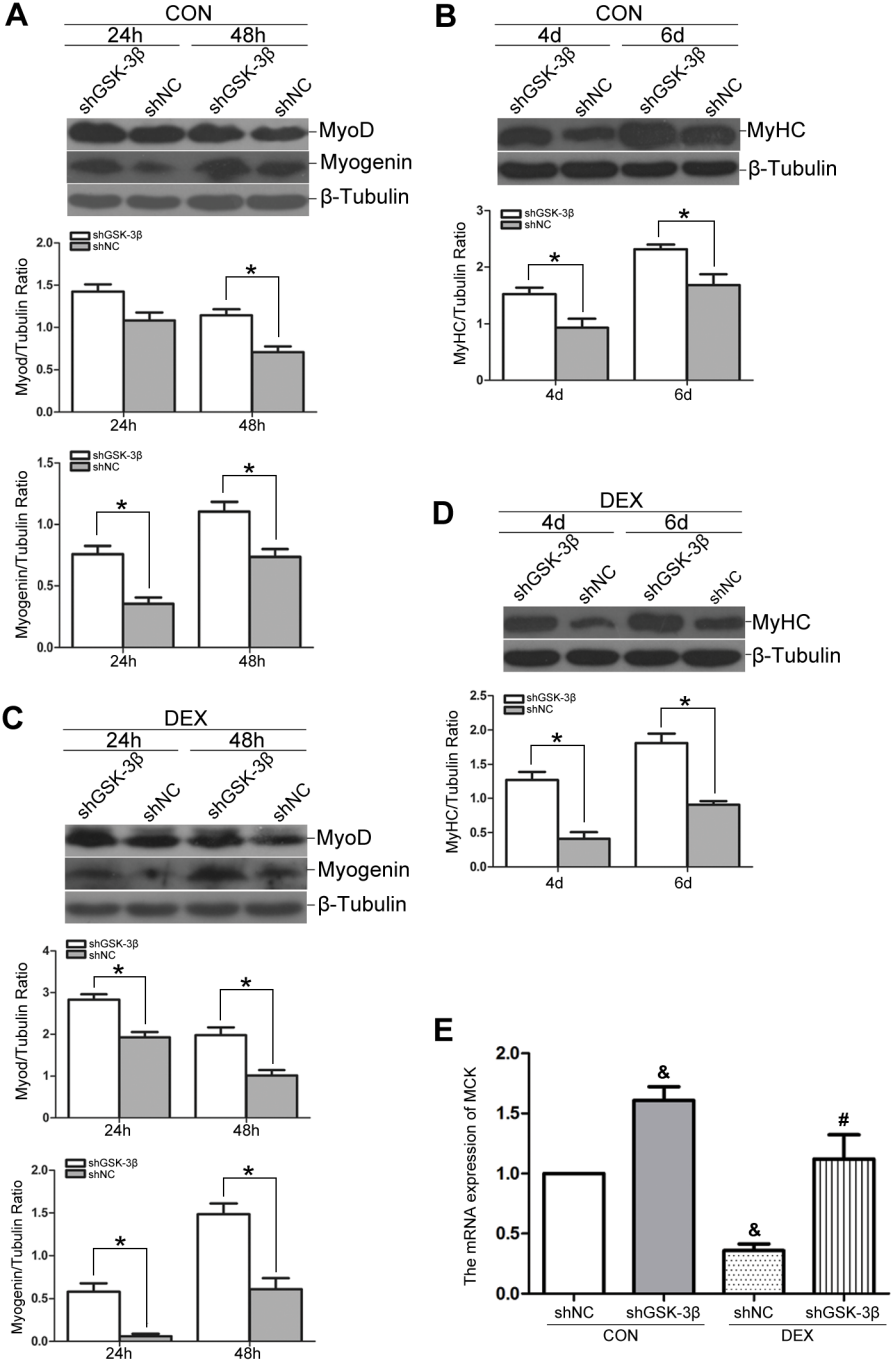
GSK-3β knockdown stimulates myogenic differentiation and confers resistance to DEX-induced inhibition of myogenic markers of differentiation. shGSK-3β and shNC myoblasts cells were induced to differentiate in the absence of DEX (CON), and Western blot analysis was used to assess the expression of MyoD and myogenin after induction for 24 h or 48 h (A) and the expression of MyHC after differentiation for 4 d or 6 d (B). shGSK-3β and shNC myoblast cells were induced to differentiate in the presence of DEX. Western blot analysis showing the expression of MyoD and myogenin after induction for 24 h or 48 h (C) and the expression of MyHC after induction for 4 d or 6 d (D). (E) MCK mRNA levels were measured in shGSK-3β and shNC myoblast cells after differentiation induction for 3 d in the absence (CON) or presence of DEX. The relative mRNA levels were assessed by real-time RT-PCR and compared with shNC myoblasts induced in control DM (without DEX). The data are shown as the means ± SEM of three independent experiments. *P<0.05; ^&^ *P*<0.05 versus shNC/CON; ^#^ *P*<0.05 versus shNC/DEX.

In parallel, Western blot analysis was performed to examine MyoD, myogenin and MyHC expression. We observed that GSK-3β knockdown enhanced the expression of these differentiation markers in the absence of DEX. It also conferred resistance to DEX-mediated reductions in MyoD and myogenin expression at 24 h and 48 h (Fig. 5A and 5C) and in MyHC expression at 4 d and 6 d (Fig. 5B and 5D) after the induction of myogenic differentiation. Finally, we examined the MCK mRNA levels in transfected cells after 3 d of differentiation. As shown in Fig. 5E, in the absence of DEX, MCK mRNA expression was up-regulated in shGSK-3β myoblasts compared with shNC myoblasts. Moreover, the DEX-mediated down-regulation of MCK mRNA expression was ameliorated by shGSK-3β knockdown, as demonstrated by the similar MCK mRNA expression between DEX-treated shGSK-3β myoblasts and control shNC myoblasts.

### GSK-3β inhibition attenuates dexamethasone-mediated repression of myogenic differentiation in primary satellite cells

**Figure 6:**
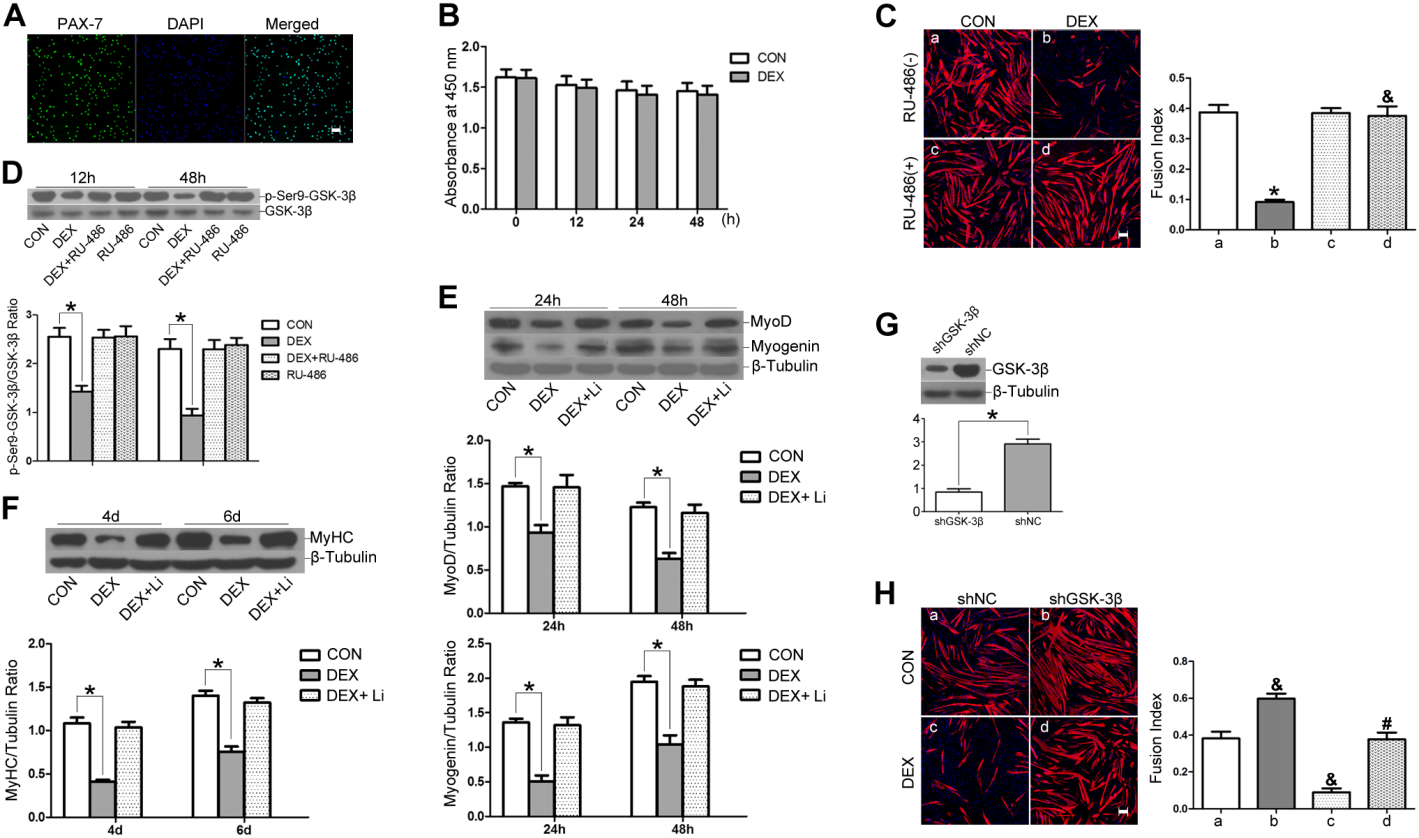
GSK-3β inhibition attenuates dexamethasone-mediated repression of myogenic differentiation of primary satellite cells. (A) Immunofluorescence analysis of PAX-7 (green) and DAPI (blue) expression in primary satellite cells. (B) Differentiating primary satellite cells were treated with 10^−5^ M DEX and harvested for cytotoxicity assays using a CCK-8 kit at 12 h, 24 h, and 48 h. (C) Myogenic differentiation assay to determine the GR specificity of DEX using RU-486. Immunofluorescence detection of MyHC (red) and DAPI (blue) was used to detect myotubes (left panel). The fusion index is shown in right panel. The scale bar is 50 µm. (*P<0.05 versus RU-486(−)/CON; ^&^ *P*<0.05 versus RU-486(−)/DEX. n = 3 independent experiments). (D) Differentiating primary satellite cells were incubated with DEX and RU-486 (10 µM), alone or in combination, or control DM (CON). Phosphorylation of GSK-3β at serine 9 at the indicated time points was determined using Western blot analysis. (E and F) Primary satellite cells were treated with DEX (10^−5^ M) or a combination of DEX and 5 mM LiCl after switching to DM. Western blot analysis of MyoD and myogenin at 24 h and 48 h (E) and MyHC at 4 d and 6 d (F). (G) The GSK-3β abundance was assessed by Western blot analysis to detect the silencing efficiency after shRNA transfection for 24 h. (H) shNC and shGSK-3β satellite cells were differentiated in the absence (CON) or presence of DEX (DEX) for 4 d. Immunofluorescence detection of MyHC (red) and DAPI (blue) were used to detect myotubes (left panel). The fusion index is shown in right panel. The scale bar is 50 µm. The data are shown as the means ± SEM of three independent experiments. *P<0.05; ^&^ *P*<0.05 versus shNC/CON; ^#^ *P*<0.05 versus shNC/DEX.

The findings obtained from C2C12 myoblasts were further verified in mouse primary satellite cells. As shown in Fig. 6A, more than 95% of the primary cells were PAX-7 positive [19]. Cytotoxicity assays using CCK-8 revealed that DEX at a concentration of 10^−5^ M had no significant toxic effect on differentiating primary satellite cells (Fig. 6B). DEX addition significantly impaired myotube formation from primary satellite cells, which was nearly rescued by RU-486 treatment, as shown by MyHC staining and the fusion index analysis (Fig. 6C).

Next, we examined the phosphorylation of GSK-3β during the induction of myogenic differentiation. GSK-3β phosphorylation at serine 9 was detected at an early stage of differentiation after incubation with DEX and RU-486 (10 µM), alone or in combination. The results displayed that DEX decreased the phosphorylation of GSK-3β on serine residue 9 via the GR (Fig. 6D), which was consistent with the effects of DEX on differentiating C2C12 myoblasts.

To test the contribution of GSK-3β to DEX-mediated repression of the myogenic differentiation of primary satellite cells, we evaluated the effects of GSK-3β inhibition on DEX-mediated repression of myogenic differentiation. We first performed pharmacological inhibition experiments using LiCl. Differentiating primary satellite cells were treated with DEX or co-treated with DEX and LiCl (5 mM), and myogenic differentiation was evaluated by Western blot analysis of the expression levels of MyoD, myogenin and MyHC. As shown in Fig. 6E and 6F, the DEX-induced decreased expression levels of MyoD and myogenin at the early stage and of MyHC at the late stage were nearly rescued by co-treatment with LiCl. Then, GSK-3β gene knockdown experiments using specific shRNA sequence were performed to further verify the role of GSK-3β. Gene knockdown efficiency was assessed by Western blot analysis, which showed a degree of knockdown of approximately 70% (Figs. 6G). After differentiation for 4 d in the absence of DEX, shGSK-3β primary satellite cells displayed robust myotube formation compared with shNC primary satellite cells using MyHC staining and fusion index analysis (Fig. 6H). Furthermore, DEX-induced impairment of myotube formation from primary satellite cells was apparently rescued by GSK-3β knockdown as shown by MyHC staining and a similar fusion index compared with shNC satellite cells in control condition (Fig. 6H).

## Discussion

Supra-physiological glucocorticoids lead to the impairment of myogenic differentiation and muscle atrophy [5], [22], [23]. GSK-3β plays a central role as a negative regulator of myogenic differentiation and is known to be involved in muscle atrophy. GSK-3β inhibition is potent enough to facilitate myogenic differentiation and prevents the proteolysis and myotube atrophy caused by glucocorticoid treatment *in vitro* [22]. The results presented in this study showed that glucocorticoids had adverse effects on myogenic differentiation and, for the first time, indicated the involvement of GSK-3β in glucocorticoid-mediated suppression of myogenic differentiation. By inhibiting GSK-3β, we show an alternative strategy for alleviating the adverse effects of glucocorticoids on myogenic differentiation.

Chronic treatment with glucocorticoids is the only effective pharmacological treatment for DMD. Yet paradoxically, chronically increased levels of glucocorticoids are well-known to induce muscle atrophy and exert anti-myogenic effects on muscle satellite cells to prevent skeletal muscle from recovering from injury. In this study, we have found that supra-physiological DEX induced the suppression of myogenic differentiation in a dose-dependent manner. Although the concentration of DEX used in our *in vitro* experiments may be considered supra-physiological, up to 10^−5^ M concentrations of DEX are actually widely used in cell culture experiments to analyze glucocorticoids' effects on myogenic differentiation [3], [5], [24]. Importantly, cytotoxicity assays revealed that DEX at a concentration of 10^−5^ M had no significant toxic effects on differentiating myoblasts, which suggested that DEX-induced suppression of myogenic differentiation mainly resulted from its interference with the myogenic differentiation process. In addition, in our study, GR specificity experiments using RU-486 demonstrated that DEX exerted its anti-myogenic effects mainly through a steroidal pathway via the GR.

Myogenic differentiation is tightly orchestrated and can be roughly divided into two stages [25]. Muscle regulatory factors (*e.g.*, MRFs, composed of Myf5, MyoD, myogenin and MRF4), which have been identified as master regulators of myogenic differentiation, are induced hierarchically in the earlier stage of differentiation, and the expression of muscle specific proteins (*e.g.*, troponin, MyHC, MCK) occurs in the late stage of differentiation [26]. Our research showed that DEX reduced the expression of the early myogenic markers MyoD and myogenin. Because of the down-regulation of the MyHC protein and MCK mRNA, the DEX-mediated suppression of myogenic differentiation may mainly be due to the impaired expression and transcriptional activity of MyoD. The minor nuclear accumulation of myogenin then results in the mild induction of muscle-specific protein expression and myoblast fusion.

In recent years, evidence has emerged that GSK-3β, which is involved in both IGF-I and Wnt/β-catenin signaling, plays a central role as a negative regulator of myogenic differentiation [12], [13], [27], [28]. These findings highlight that inactive GSK-3β was indispensable for myogenic differentiation after withdrawal from the cell cycle. In this study, we showed that the phosphorylation of GSK-3β at serine 9, which results in an inactive form of GSK-3β, was gradually decreased in DEX-treated myoblasts compared to control myoblasts, and this effect was ablated by RU-486. This result suggests an activating effect of DEX on GSK-3β activity via the GR. Given the negative effect of GSK-3β on myogenic differentiation, we hypothesized that GSK-3β might be implicated in the DEX-induced suppression of myogenic differentiation. The association of GSK-3β activity with severe glucocorticoid-induced side effects has been found in multiple biological processes, including myotube atrophy [22], bone loss and the suppression of hippocampal neurogenesis under chronic stress [16], [17], [29]. To our knowledge, the involvement of GSK-3β in the glucocorticoid-induced suppression of myogenic differentiation has not been proposed.

In support of our hypothesis, the results showed that the pharmacological inhibition of GSK-3β with LiCl could attenuate the adverse effect of DEX on myogenic differentiation. LiCl inhibits GSK-3β by increasing the phosphorylation of serine 9 of GSK-3β, and this inhibitor has been widely used to determine the role of GSK-3β in various biological processes [12], [20], [30], [31]. In the current study, we noted that 5 mM LiCl was sufficient to rescue the myogenic differentiation of glucocorticoid-stressed myoblasts, as demonstrated through enhanced MyHC protein expression, MCK mRNA expression and increased myotube formation. Moreover, we employed genetic knockdown as another independent model to specifically inhibit GSK-3β. Consistent with a previous study, the genetic inhibition of GSK-3β improved myogenic differentiation by enhancing myotube formation and up-regulating the expression of MyoD, myogenin, MyHC and MCK [13]. We noted that both GSK-3β knockdown and the LiCl-based pharmacological inhibition of GSK-3β conferred resistance to the DEX-induced suppression of myogenic differentiation.

We first postulated that the enhanced myogenic differentiation induced by GSK-3β inhibition might be due to improved myoblast survival because GSK-3β played a crucial role in mediating the protective anti-apoptotic effect of M-cadherin during differentiation [32]. However, we ruled out this possibility when we found no differences in the total nuclear count after 4 d of differentiation and when an apoptosis assay analyzing cleaved caspase-3 showed no significant difference in regards to myoblast survival in our *in vitro* system (Fig. S1); this result was consistent with that of a previous study [12]. Previous reports have demonstrated that LiCl has the ability to mimic Wnt/β-catenin signaling [12], [33], which was shown to precede MRFs expression [34] and that the inhibition of GSK-3β by LiCl recruited reserve myoblasts into the differentiation process [27], [28]. The data from our study showed that GSK-3β inhibition enhanced MyoD and myogenin expression. Therefore, the activation and recruitment of reserve myoblasts and enhanced transcriptional activation of MRFs might lead to robust myogenic differentiation.

During myogenic differentiation, various differentiation-promoting signals are delivered through GSK-3β, which might regulate myogenic differentiation by targeting multiple substrates. Nuclear factor of activated T-cells c3 (NFATc3) is one target of GSK-3β. GSK-3β together with calcineurin determines the transcriptional activity of NFATc3. Calcineurin stimulation dephosphorylates NFATc3, resulting in its nuclear translocation. However, GSK-3β counteracts this process via inhibitory phosphorylation. Previous studies have demonstrated that GSK-3β-deficient myoblasts displayed an enhanced nuclear translocation of NFATc3 and elevated NFAT-sensitive promoter transactivation, which led to increased muscle gene promoter transactivation and enhanced myotube formation [13]. Furthermore, the inactivation of GSK-3β in myoblasts led to the nuclear accumulation of β-catenin, a crucial downstream effector molecule in Wnt signaling, and increased β-catenin-dependent transcriptional activity to promote myoblast fusion [14], [35]. Further studies are required to identify the downstream target for GSK-3β that is responsible for glucocorticoid-mediated suppression of myogenic differentiation.

In summary, the present study strengthens the concept that GSK-3β plays a critical role in myogenic differentiation and demonstrates that inhibiting GSK-3β signaling attenuates glucocorticoid-mediated myogenic differentiation suppression. Our data indicate that GSK-3β inhibition could be a potential strategy for the prevention or treatment of glucocorticoid-induced muscle deterioration, but this should be confirmed in future studies.

## Supporting Information

Figure S1 **LiCl showed no significant protective effects on differentiating myoblasts.** Differentiating C2C12 myoblasts and primary satellite cells (SC) were treated with or without 5 mM LiCl for 4 d and then labeled with DAPI (100×) (A) or processed for immunoblotting of cleaved caspase-3 (B).
(TIF)
