# Supplementary figures and images for: Inhibition of Glycogen Synthase Kinase-3β Attenuates Glucocorticoid-Induced Suppression of Myogenic Differentiation In Vitro
Source: PLoS One. 2014 Aug 15;9(8):e105528. doi: 10.1371/journal.pone.0105528 (PMC4134315; doi:10.1371/journal.pone.0105528)

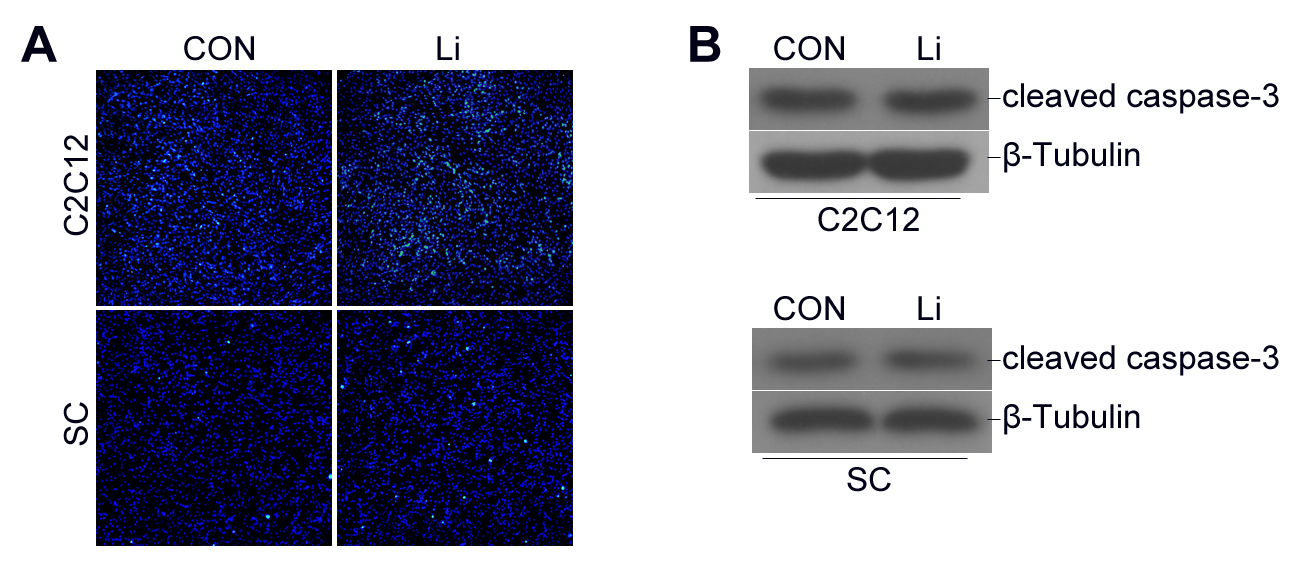

Supplement: Figure S1 — LiCl showed no significant protective effects on differentiating myoblasts. Differentiating C2C12 myoblasts and primary satellite cells (SC) were treated with or without 5 mM LiCl for 4 d and then labeled with DAPI (100×) (A) or processed for immunoblotting of cleaved caspase-3 (B). (TIF) [file pone.0105528.s001.tif]
